# Seminal parameters of patients with Type 2 Diabetes Mellitus undergoing Assisted Reproduction: A retrospective analysis

**DOI:** 10.5935/1518-0557.20250044

**Published:** 2025

**Authors:** Gabriel Acácio de Moura, Mayara Lobato Lourenço, João Pedro Viana Rodrigues, Yasmim Mendes Rocha, Hamilton de Martin, Eduardo Gomes Sá, Sebastião Evangelista Torquato, Eduardo de Paula Miranda, Roberto Nicolete

**Affiliations:** 1 Oswaldo Cruz Foundation (FIOCRUZ), Eusébio-CE, Brazil; 2 Sollirium Educational Center for Reproductive Medicine, Fortaleza-CE, Brazil

**Keywords:** type II diabetes mellitus, semen analysis, metabolic diseases, fertility, reproductive techniques, male fertility

## Abstract

**Objective:**

This study aims to investigate the impact of T2DM on the seminal parameters of patients undergoing assisted reproduction techniques.

**Methods:**

A retrospective analysis of medical records was conducted at the at our institution from January 2017 to January 2022, including data on patients age, presence of Type 1 or T2DM, prediabetes, and seminal parameters such as concentration, Kruger morphology, motility, pH, ejaculate volume, and sperm DNA fragmentation.

**Results:**

Seminal parameters of patients with T2DM did not show significant differences compared to patients with normal controls. Conversely, evaluation of HbA1c as a continuous variable in our linear regression model indicated that this parameter was associated with elevated sperm DNA fragmentation.

**Conclusions:**

T2DM was not found to be a determining factor in the deterioration of overall seminal quality in patients undergoing assisted reproduction techniques, with a potential negative effect on sperm DNA fragmentation.

## INTRODUCTION

Diabetes Mellitus (DM) is delineated as a multifaceted metabolic disorder characterized by chronic hyperglycemia stemming from deficiencies in insulin action or production (Fan *et al*., 2022). Of particular interest is Type 2 Diabetes Mellitus (T2DM), the most prevalent form of the disease, accounting for approximately 95% of diabetes cases worldwide, as reported by the World Health Organization (WHO). This condition is marked by a functional impairment of the β-pancreatic cells, leading to ineffectiveness in insulin production and, consequently, elevated blood glucose levels, laying the groundwork for a plethora of metabolic complications (Padhi *et al*., 2020).

One mechanism through which T2DM exerts adverse effects is via oxidative stress, induced by an imbalance between Reactive Oxygen Species (ROS) production and the body’s antioxidant defense. This phenomenon has direct implications for the male reproductive system, affecting structures such as the hypothalamic-pituitary-gonadal axis, testicles, and accessory glands, potentially culminating in male infertility scenarios (Dai *et al*., 2022). Furthermore, studies indicate that DM has a notable impact on the hormonal function and seminal parameters of affected individuals (Ali *et al*., 2022), with evidence also suggesting an association between T2DM and erectile dysfunction. However, the underlying mechanisms remain to be fully elucidated (Zhu *et al*., 2023).

Given the high prevalence of T2DM and its potential implications for male reproductive health, it becomes imperative to deepen our understanding of its effects on patients turning to Assisted Reproductive Technologies (ART) for conception. Understanding these impacts could offer valuable insights for optimizing treatments and counseling strategies in specialized centers, particularly in regions with a significant incidence of this comorbidity, such as Fortaleza-Ceará (Moura & Monteiro, 2020; Li *et al*., 2023). Therefore, through a retrospective cohort, this study proposes to investigate the role of T2DM and prediabetes in the seminal parameters of patients undergoing ARTs, aiming to elucidate their influence on male fertility and support more effective clinical practices.

## MATERIALS AND METHODS

### Study Type

The investigation constitutes a retrospective cohort study that analyzes clinical records of patients who underwent Assisted Reproduction Technologies (ART) at the Evangelista Torquato Human Reproduction Center in Fortaleza - Ceará, Brazil. The study period, from January 1st, 2017, to January 1st, 2022, was chosen to encompass a representative time frame before and during the implementation of new clinical guidelines at the institution. The selection of records utilized the digital platform gsDOCTOR, which compiles comprehensive clinical information, including treatment outcomes and biochemical analyses. This study was conducted by the ethical guidelines of the Human Research Ethics Committee, approved under protocol No. 58462022.8.0000.5054.

### Patients

Data were collected from patients undergoing seminal analysis for ART procedures for all cycles included in the study. For this study, inclusion and exclusion criteria of records were adopted to optimize sample selection. The inclusion criteria accepted for the study were all couples undergoing ART, where the male partner had a history of Type 2 Diabetes Mellitus (T2DM) and prediabetes (as per glycosylated hemoglobin parameters), in addition, records of couples where seminal parameters followed the WHO normality standards were used as a control group. Exclusion criteria eliminated records of couples where the male partners had a history of comorbidities leading to infertility or records containing incomplete patient information.

### Diabetes and Glycosylated Hemoglobin Measurement

Patient groups for the current study were segregated based on the results observed in the analyzed records regarding the glycosylated hemoglobin (HbA1c) test. The reference values used for screening records were within a range of up to 5.7% for patients without comorbidity, above 6.5% for patients with T2DM (without a previous diagnosis of Type 1 Diabetes Mellitus) and within a range of 5.8 to 6.4% for prediabetic patients.

### Data Collection

After selecting the eligible records based on the aforementioned inclusion and exclusion criteria, the following data were collected from the gsDOCTOR platform for tabulation: Patient age, presence or absence of Diabetes Mellitus Type 1 or T2DM and prediabetes, and seminal quality parameters including concentration (CT), Kruger morphology (MK), Motility (MT), pH, ejaculate volume, and sperm DNA fragmentation.

### Statistical Analysis

All quantitative data were represented as mean±standard deviation. The Shapiro-Wilk test was used to analyze the normality of sample distribution due to the sample size. In cases of normal distribution, data were compared using the ANOVA test, and for non-normal distributions, the Kruskal-Wallis non-parametric test was applied. Simple linear regression with an R^2^ >0.07 was conducted to achieve a better correlation among parameters. A significance level of *p*<0.05 was adopted for the tests. The software GraphPad Prism (Version 6) was used for all statistical analyses.

## RESULTS

### Demographic Data

A total of 222 records were evaluated, of which 117 were from patients without any identifiable male infertility factors, 79 records were from prediabetic patients, and 26 were from patients with confirmed T2DM. The HbA1c rate in the control group was around 5.06±0.20%, in the prediabetic patient group 5.73±0.33%, and in patients with T2DM 8.18±2.34%. The age of patients in the control, prediabetic, and T2DM groups ranged between 37.34±6.81, 38.78±8.19, and 46.00±8.75, respectively. All data are available in Table 1.

**Table 1:** Seminal Parameters and Demographic Profile of Patients Eligible for the Study.

Parameter	Control	Prediabetics	T2DM	*p*-value
**n**	117	79	26	-
**Glycated Hemoglobin HbAc1 (%)**	5.06±0.20	5.73±0.33	8.18±2.34	-
**Age**	37.34±6.81	38.78±8.19	46.00±8.75	-
**Concentration (1x106)**	37.46±34.19a	38.97±41.42	48.52±37.81	0.50
**Motility (%)**	41.92±19.00	39.24±18.36	35.10±19.21	0.26
**Kruger Morphology**	6.04±4.23	8.28±6.60	6.35±3.71	0.06
**Ph**	8.00±0.86	8.14±0.41	8.28±0.51	0.22
**Volume (mL)**	2.85±1.40	2.94±1.62	3.56±3.31	0.97
**Sperm DNA Fragmentation (%)**	21.52±6.81	18.45±5.28	24.43±6.45	0.10

Legend: Type 2 Diabetes Mellitus - T2DM; Glycated Hemoglobin - HbA1c; For *p*-value <0.05 - (^a^).

### Prediabetic Patients and Seminal Parameters

Prediabetic patients showed no significant difference in sperm concentration levels compared to the control group (37.46±34.19; 38.78 ± 8.19; *p*>0.05). Sperm motility was another parameter unaffected in the prediabetic patient group compared to the control group (41.92±19.00; 39.24±18.36; *p*>0.05). The Kruger morphology parameter also did not show a statistical difference compared to the control group (6.04±4.23; 8.28±6.60; *p*>0.05). Regarding sperm pH, no significant difference was observed between the groups (8.00±0.86; 8.14±0.41; *p*>0.05), the same pattern was observed in volume (2.85±1.40; 2.94±1.62; *p*>0.05) and sperm DNA fragmentation.

### Patients with Type 2 Diabetes Mellitus and Seminal Parameters

For patients with T2DM, no statistical differences were observed in sperm concentration levels compared to the control group (37.46±34.19; 48.52±37.81; *p*>0.05). Sperm motility also did not present significant changes compared to the control group (41.92±19.00; 35.10±19.21; *p*>0.05). Kruger morphology showed no significant difference with the control group (6.04±4.23; 6.35±3.71; *p*>0.05). Meanwhile, pH (8.00±0.86; 8.28±0.51; *p*>0.05), volume (2.85±1.40; 3.56±3.3; *p*>0.05), and sperm DNA fragmentation (21.52±6.81; 24.43±6.45; *p*>0.05) did not show a significant difference when compared to the control group of patients.

### Patients with Type 2 Diabetes Mellitus *vs*. Pre-Diabetics

Comparing seminal parameters of pre-diabetic patients with those with T2DM, we observed no statistical difference in sperm concentration (38.97 ± 41.42; 48.52 ± 37.81, *p* > 0.05). The same was observed in the sperm motility parameters of the pre-diabetic and T2DM groups (39.24 ± 18.36; 35.10 ± 19.21, *p* > 0.05) and Kruger morphology (8.28 ± 6.60; 6.35 ± 3.71, p> 0.05). The pH (8.14±0.41; 8.28±0.51 *p* > 0.05) and volume (2.94±1.62; 3.56±3.31 *p* > 0.05) parameters of both groups did not differ statistically. While the levels of sperm DNA fragmentation, although slightly elevated, did not show statistical differences between the groups (18.45±5.28; 24.43±6.45 *p* > 0.05).

### Serum HbA1c Levels and Seminal Parameters

**Figure 1:**
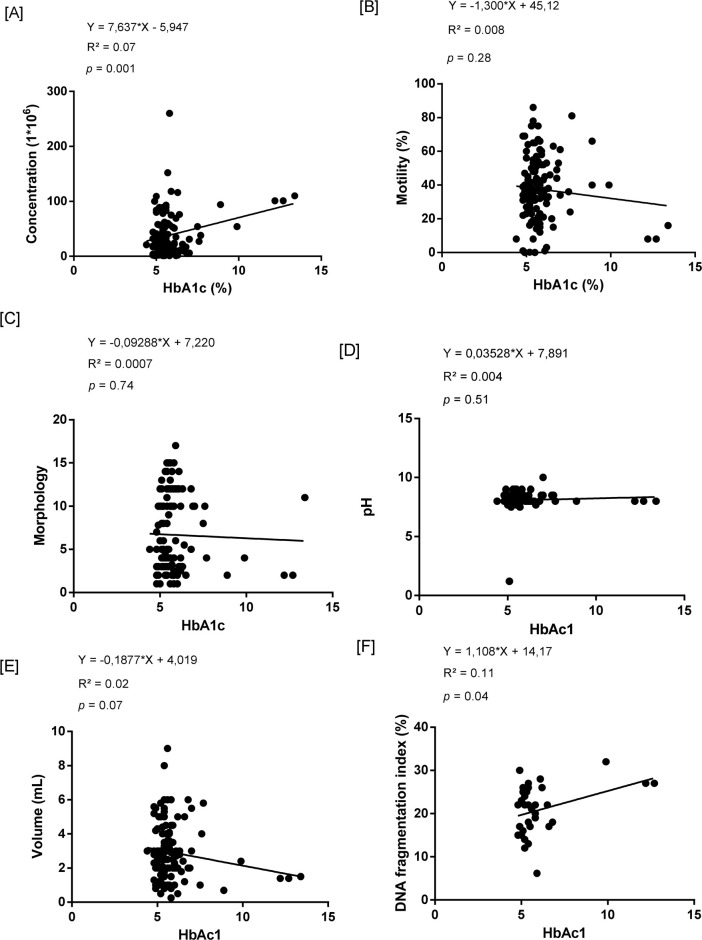
Simple Linear Regression Model of HbA1c Against Seminal Parameters. Legend: (A) Glycated hemoglobin index versus sperm concentration levels, (B) Motility, (C) Morphology, (D) pH, (E) Volume, and (F) Sperm DNA Fragmentation. HbA1c (Glycated Hemoglobin); *p*-value>0.05.

A simple linear regression analysis was performed to correlate seminal parameters of patients undergoing ARTs with HbA1c levels, as shown in the graphs in Figure 1. These data showed that an increase in HbA1c was associated with an increase in sperm concentration and sperm DNA fragmentation, as shown in Figures 1A and 1F, respectively (0.001; 0.04; *p*<0.05). However, our linear regression model presented an R^2^ in the three parameters of (0.07; 0.11; R^2^ < 0.07), indicating that although there is a correlation between the variables, the model may not be precise enough to predict the activity of HbA1c levels on sperm concentration parameters and sperm DNA fragmentation.

## DISCUSSION

Diabetes Mellitus (DM) is not merely characterized as a singular disease; rather, it encompasses a group of conditions unified by a primary diagnostic criterion - hyperglycemia. This convergence leads to a spectrum of metabolic disorders, among which Type 2 Diabetes Mellitus (T2DM) currently represents the predominant category, accounting for 90 - 95% of cases (Cole & Flores, 2020). Furthermore, data from the World Health Organization (WHO) have indicated a significant increase in the global diabetes population, soaring from 108 million in 1980 to 422 million in 2014, with a particularly sharp rise in lowand middle-income countries (WHO, 2024). Although substantial progress has been made in understanding and treating T2DM, its prevalence, and associated morbidity and mortality rates continue to escalate, underscoring the urgent need for enhanced screening and monitoring strategies to mitigate future complications for affected individuals (He *et al*., 2021).

Reproductive dysfunction and a decline in semen quality have been documented in association with conditions such as obesity and diabetes. However, the molecular signaling pathways and their exact impact on male reproductive health remain ambiguous (AbbasiHormozi *et al*., 2023). Additionally, pre-clinical studies have suggested that hyperglycemia may predispose individuals to urogenital infections and neurological disorders, which could, in turn, significantly affect seminal parameters and fertility (Facondo *et al*., 2022). Thus, a deeper comprehension of the impact of this comorbidity on male fertility could substantially inform decision-making processes in assisted reproductive technologies (ART), potentially improving clinical outcomes (Moura & Monteiro, 2020).

This study did not identify significant alterations in the seminal parameters of patients with T2DM compared to control patients undergoing assisted reproduction treatment. These findings diverge from the existing literature. In a previous clinical study, AbbasiHormozi *et al*. (2023) examined the potential impact of T2DM on normal and obese diabetic patients and control groups without diabetes, finding that parameters such as progressive motility, sperm concentration, and morphology were significantly lower in the T2DM group compared to the control group. Furthermore, a clinical study by Condorelli *et al*. (2018) demonstrated impaired concentration levels, progressive motility, and morphology in patients with Type 1 and Type 2 Diabetes Mellitus compared to control patients.

This discrepancy may be linked to the prior identification and treatment of T2DM patients, which could contribute to improvements in their seminal parameters. A pre-clinical study by Soliman *et al*. (2019) managed to mitigate sperm concentration, motility, and viability parameters in mice through nine weeks of treatment with olive extracts compared to a standard control group. Moreover, a review by Shpakov (2021) reported that the use of the drug metformin enhances lipid and carbohydrate metabolism, as well as insulin sensitivity, thereby preventing the overproduction of reactive oxygen species and pro-inflammatory factors in men with T2DM, which could have a restorative effect on the reproductive functions of patients with these metabolic disorders.

Our research also disclosed that prediabetic patients did not exhibit significant differences in seminal parameters such as sperm concentration, Kruger morphology, motility, pH, volume, and sperm DNA fragmentation compared to control group patients. This association might be related to levels within an acceptable normal range for the body, not causing a metabolic disorder. Furthermore, as these are patients requiring assisted reproductive technologies (ART), they receive medical monitoring concerning treatment to enhance biochemical parameters, aiming to optimize outcomes. This observation aligns with the study by Krysiak *et al*. (2021), which found that metformin treatment in prediabetic patients regulated gonadotropin levels, directly linked to gamete production.

However, although we did not identify a robust predictive model regarding concentration, our linear regression analysis observed an increase in this parameter. A previous study carried out by Yannasithinon & Iaamsaard (2019) observed that in mice induced with diabetes, there was an increase in seminal vesicles and weight, which could directly influence the seminal concentration of semen. The data regarding seminal parameters and HbA1c levels differ from the study by García-Díez *et al*. (1991), which found that insulin levels in blood or seminal plasma were not correlated with seminal parameters. As mentioned previously, this discrepancy may be linked to the patients’ prior treatment, which could have improved their clinical status and, consequently, fertility.

Another critical finding was increased sperm DNA fragmentation levels in line with elevated glycosylated hemoglobin levels. In situations of bodily homeostasis, glucose uptake is mediated through the production of hydrogen peroxide and other free radicals, such as reactive oxygen species (ROS) and reactive nitrogen species (RNS), which partially overlap the glucose uptake pathways in muscle contraction or energy-generating activities (Llanos & Pallomero, 2022). In cases of insulin resistance, this mechanism aims to regulate energy production, releasing reactive species. However, accumulating these compounds results in oxidative modification of lipids, proteins, and DNA, causing errors in transcription processes and the production of cellular metabolic products (McKeegan *et al*., 2021).

According to some studies in the literature, this fact is intrinsically linked to the reduction in seminal and reproductive quality. A study by Tsounapi *et al*. (2017) using diabetic murine models to investigate the deleterious effects of T2DM observed a reduction in the seminal vesicle size of these animals compared to control groups due to lipid peroxidation caused by reactive species. Moreover, another concerning factor is that (Bassey *et al*., 2019) found that antioxidant levels were reduced in patients with T2DM compared to control groups. Both studies could corroborate the potential activity of reactive species on seminal parameters, including sperm DNA fragmentation.

The primary limitations of our study include the small number of T2DM patients, given that it is a relatively restricted population. Because the study was conducted in a single reproductive medicine center, it resulted in a low patient count. Additionally, as previously noted, the prior monitoring and effective treatment of T2DM patients could have improved or mitigated the impact of T2DM on seminal parameters, potentially introducing bias into the study as it is a retrospective analysis. On the positive side, our study stands out for conducting analyses using prediabetic patients, a seldom-used approach, and using control and prediabetic groups allows for a broader perspective on the impact of hyperglycemia on the seminal parameters of T2DM-diagnosed patients. Moreover, excluding patients with comorbidities that impair fertility may yield more robust results concerning the actual impact of T2DM.

Therefore, T2DM did not demonstrate a significant impact on the seminal parameters of patients utilizing assisted reproduction centers compared to patients with proven fertility. Additionally, we observed that parameters such as concentration, motility, pH, volume, and sperm DNA fragmentation remained unchanged in prediabetic patients. However, further studies are warranted to confirm these hypotheses

## Abbreviations:

Reactive Oxygen Species = (ROS)

Reactive Nitrogen Species = (RNS)

Diabetes Mellitus = DM

Type 2 Diabetes Mellitus = (T2DM)

Reactive Oxygen Species = (ROS)

Glycated Hemoglobin = (HbA1c)

World Health Organization = (WHO)

Assisted Reproduction Technologies = (ART).
